# Identifying intentional injuries among children and adolescents based on Machine Learning

**DOI:** 10.1371/journal.pone.0245437

**Published:** 2021-01-20

**Authors:** Xiling Yin, Dan Ma, Kejing Zhu, Deyun Li

**Affiliations:** Department of Public Health and Health Research, Center for Disease Control and Prevention of Zhuhai City, Zhuhai, Guangdong, China; Gachon University Gil Medical Center, REPUBLIC OF KOREA

## Abstract

**Background:**

Compared to other studies, the injury monitoring of Chinese children and adolescents has captured a low level of intentional injuries on account of self-harm/suicide and violent attacks. Intentional injuries in children and adolescents have not been apparent from the data. It is possible that there has been a misclassification of existing intentional injuries, and there is a lack of research literature on the misclassification of intentional injuries. This study aimed to discuss the feasibility of discriminating the intention of injury based on Machine Learning (ML) modelling and provided ideas for understanding whether there was a misclassification of intentional injuries.

**Methods:**

Information entropy was used to determine the correlation between variables and the intention of injury, and Naive Bayes (NB), Decision Tree (DT), Random Forest (RF), Adaboost algorithms and Deep Neural Networks (DNN) were used to create an intention of injury discrimination model. The models were compared by comprehensively testing the discrimination effect to determine stability and consistency.

**Results:**

For the area under the ROC curve with different intentions of injuries, the NB model was 0.891, 0.880, and 0.897, respectively; the DT model was 0.870, 0.803, and 0.871, respectively; the RF model was 0.850, 0.809, and 0.845, respectively; the Adaboost model was 0.914, 0.846, and 0.914, respectively; the DNN model was 0.927, 0.835, and 0.934, respectively. In a comprehensive comparison of the five models, DNN and Adaboost models had higher values for the determination of the intention of injury. A discrimination of cases with unclear intentions of injury showed that on average, unintentional injuries, violent attacks, and self-harm/suicides accounted for 86.57%, 6.81%, and 6.62%, respectively.

**Conclusion:**

It was feasible to use the ML algorithm to determine the injury intention of children and adolescents. The research suggested that the DNN and Adaboost models had higher values for the determination of the intention of injury. This study could build a foundation for transforming the model into a tool for rapid diagnosis and excavating potential intentional injuries of children and adolescents by widely collecting the influencing factors, extracting the influence variables characteristically, reducing the complexity and improving the performance of the models in the future.

## Background

Injuries can be classified as unintentional or intentional injuries. Intentional injuries include violent attacks and self-harm/suicide. Children and adolescents are high-risk populations for injuries. Violent attacks and abuse against children and adolescents often involve covert crimes; self-harm/suicide is often related to adverse childhood experiences (ACEs) [1, 2]. Violent attacks and self-harm/suicide represent higher disease burdens in adolescents than in other age groups [3]. In recent years, intentional injuries have also received widespread attention, as Chinese media outlets have reported that children and adolescents have increased physical violence, school bullying, self-harm/suicide and other issues. The difference in injury intentions can help us better understand the different types of injury mechanisms, facilitate the development of interventions and advance prevention work. However, the distinction between intentional and nonintentional injuries is not always easy or effective.

Research based on data estimates has shown that at least half of children in Asia, Africa and North America experienced violence in the past year [4]. A meta-analysis of global data found that child sexual abuse was 30 times higher and that physical abuse was 75 times higher than in official reports [5, 6]. Studies of Chinese children and adolescents showed that the overall prevalence of suicidal ideation and attempts was 16.10% and 3.60%, respectively; 13.20% reported having been threatened or injured by violence in schools [7]. A South Korean study reported that 10.50% of teen injuries reported by outpatient/emergency rooms were intentional injuries [8]. Amanullah S et al. [9] reported that 10.00% of children who were injured in the school environment were injured intentionally based on the National Electronic Injury Monitoring System. In many countries, the true severity of intentional injury problems is greatly underestimated. On the one hand, there is the possibility of underreporting or misreporting because data often come from passive reporting by health systems; on the other hand, there may be some misclassification of injury intentions for various other reasons. Compared with other studies, injury monitoring from outpatient/emergency rooms among Chinese children and adolescents captured low levels of intentional injury (4.84%) [10]. Doctors play a key role in identifying and preventing the recurrence of intentional injuries. However, because unintentional and intentional injuries have many common mechanisms of injury, this distinction can be very challenging for doctors [11]. For example, road traffic injuries may be unintentional injuries, or they may involve a violent attack or self-harm/suicide. In practice, doctors usually make judgements by asking patients or guardians for information to inform the clinical diagnosis, but this information can be subjective. Although the distinction between intentional and unintentional injury is valuable, the commonality to the subfields makes the distinction difficult. This intent distinction is not always easy or effective. It does not rule out the existence of misclassification of intentional injuries.

The study of the misclassification of intentional injuries in the literature is relatively lacking. Machine Learning (ML) is an interdisciplinary subject developed in recent years and is the core of artificial intelligence. To date, no relevant study reports using the ML algorithm to judge the intention of injury have been offered.

This study intended to understand whether there was a misclassification of intentional injuries, to explore the actual level of intentional injury by extracting the characteristics of injury cases among children and adolescents from outpatient/emergency rooms, to calculate the contribution of the independent variable classification of intention of injury, and to use Naive Bayes (NB), Decision Tree (DT), Random Forest (RF), Adaboost algorithms and Deep Neural Networks (DNN) to model the discrimination between intentional and unintentional injury to screen for the best model. This study was expected to objectively discriminate between intentional and unintentional injury through the model, to reduce the false positive rate of unintentional injury, and to effectively avoid the wrong classification; meanwhile, this study aimed to identify the potential intentional injuries of children and adolescents through discrimination models.

## Methods

### Data collection

Cases aged 0–17 who were diagnosed as injured were used from the Chinese National Injury Surveillance System (NISS) in Zhuhai City, China, from January 1, 2006, through December 31, 2017. NISS collected injury cases on the initial visits for all injuries in emergency rooms and outpatient clinics in 3 sentinel hospitals.

There were three types of intents resulting in injuries. Unintentional injury meant an injury due to accidental events. Self-harm/suicide refers to injuries inflicted by the patient who was known to be injured, either directly or indirectly, by some positive or negative action that could have resulted in injury or death. Violent attacks meant the patient had been deliberately attacked or violently injured by another person. Either self-harm/suicide or violent attack was classified as intentional injury.

### Ethics statement

This study protocol had been approved by the Ethics Committee of Center for Disease Control and Prevention in Zhuhai, and followed the tenets of the Declaration of Helsinki. When the doctor treated the injured patient, it was accompanied by the data collection process, with the nurse and the patient’s guardian at the scene. The patient’s age, gender, time and location of the injury were included in the general medical record of the consultation. Verbal informed consent was obtained from parents whose children and adolescents aged <18 years after the nature of the study had been explained. Our research would not cause damage to the patients, and the personal privacy of the patient’s name, address, contact information, and so on was not involved in the research. The verbal informed consent instead of written consent obtained from the children’s guardian had been approved by the ethics committee.

### Analysis

Using R 3.6.0 to model and identify the intentions of injury, the main packages were called “rpart”, “rpart.plot”, “randomForest”, “adabag”, “e1071”, “DMwR”, “ROCR”, “nnet”.

#### Data pre-processing and characterization

The Educational level and nature of the injury contained one missing value, which was filled with the majority. The epidemiological characteristics of the factors were described from the perspective of the intention of injury classification. It was calculated to characterize the differences between different intentional injuries (including age group, gender, region, time of injury, etc.), and using a χ^2^ or fisher exact test, p < 0.05 was considered statistically significant.

#### Calculate the contribution of the predictor

Using information entropy to determine the correlation between variables and injury intentions (calculating the contribution of predictors), the purpose was to understand the correlation between discrete features, to filter out variables that were unmeaningful to the classification, and to reduce redundant features. The correlation between discrete features was described by the information gain and information gain ratio in the classic algorithm of the decision tree. Some of the "non-discriminative" variables were removed from the model. The predictive variables with a contribution index <0.007 were removed from the model. On the one hand, the efficiency of the modelling program was improved, and on the other hand, when the model was built using all variables, it was found that the variables did not participate in the final prediction.

#### Training\Building model

Data on the intention of injury = 1/2/3 (1 = unintentional injury, 2 = self-harm/suicide, 3 = violent attack) were used for training. K folds cross-validation method (5 folds) was used to train the data set. Since the data imbalance (proportion of intention = 1 >90.00%) caused the characteristics of the sample with sparse samples to be insufficiently learned, and the prediction effect deteriorated, it was improved by resampling in the training set.

NB, DT, RF, Adaboost algorithms and DNN were applied to establish a classification model and to provide model evaluation indicators such as false positive probability confusion matrix, recall, precision, and accuracy. In addition, for unbalanced data sets, it was necessary to investigate the F1-score value. The Receiver Operating Characteristic (ROC) curve and the area under the curve were drawn and calculated.

Confusion matrix:

$$\begin{array}{c} \begin{array}{c} \text{Schematic}\;\text{table}\;\text{of}\;\text{Confusion}\;\text{matrix}\\ \begin{array}{cc} \text{Predictive}\;\text{value} & \begin{array}{c} \text{Actual}\;\text{value}\\ \begin{array}{cc} \text{Positive} & \text{Negative} \end{array} \end{array}\\ \begin{array}{c} \text{Positive}\\ \text{Negative} \end{array} & \begin{array}{cc} \text{TP}\; & \;\text{FP}\\ \text{FN}\; & \;\text{TN} \end{array} \end{array} \end{array} \end{array}$$

Formula of index calculation:

$$\text{Recall}=\frac{\text{TP}}{\text{TP}+\text{FN}}$$

$$\text{Precision}=\frac{\text{TP}}{\text{TP}+\text{FP}}$$

$$\text{Accuracy}=\frac{\text{TP}+\text{TN}}{\text{TP}+\text{FN}+\text{FP}+\text{TN}}$$

$$\text{F1}-\text{score}=\frac{\text{2}\times (\text{Recall}\times \text{Precision})}{\text{Recall}+\text{Precision}}$$

#### Model test

The data with intention of injury = 4/5 (4 = unclear, 5 = others) was used to test whether the prediction of different algorithm data was stable and consistent. The result of the comparison between the two algorithms was output.

#### Model parameters setting

Model parameters were set as follows. NB: resample = TRUE, samplerate = c(1,4,2). DT: minsplit = 10, minbucket = 150, cp = 0.00017, resample = TRUE, samplerate = c(1,20,2). RF: ntree = 100, mtry = 4, resample = TRUE, samplerate = c(1,20,2). Adaboost: mfinal = 10, resample = TRUE, samplerate = c(0.5,10,1). DNN: size = 6, rang = 0.1, decay = 5e-1, maxit = 200, resample = TRUE, samplerate = c(1,20,2). Other parameters were set to the default settings in the packages.

## Results

### Epidemiological characteristics of injury intentions

Among the 86 389 cases of children and adolescents ≤17 years old from 2006 to 2017, 81,459 cases (94.29%) were judged as unintentional injuries by clinicians, 4218 cases (4.88%) were intentional injuries, and 712 cases (0.83%) were others / not clear. Among the intentional injuries, 4013 cases (95.14%) were violent attacks, and 205 cases (4.86%) were self-harm/suicide.

Unintentional injuries, violent attacks and self-harm/suicide among children and adolescents in different regions, of different genders, with different household registrations, and with different education levels and so on had different numbers of injuries (p<0.001). There was no difference in whether the injury occurred on weekdays/weekends (χ^2^ = 1.92, p = 0.383). Falls accounted for 50.37% of unintentional injuries, followed by animal bites (13.81%) and road traffic injuries (12.02%). Blunt injuries accounted for 59.43% of violence/attacks. Knife/sharp injuries and poisoning accounted for 37.07% and 23.41% of self-injury/suicide, respectively (Table 1).

**Table 1:** Number of cases and composition ratio of unintentional and intentional injuries among children and adolescents aged 0–17 in Zhuhai City from 2006 to 2017.

		Unintentional injuries n (%)	Intentional injuries		Total n (%)	*χ*^*2*^	*P*
			Violent attacks n (%)	Self-harm / suicide n (%)			
Region	Urban	58092 (71.31)	2307 (57.49)	129 (62.93)	60528 (70.65)	358.48	<0.001
	Rural	23367 (28.69)	1706 (42.51)	76 (37.07)	25149 (29.35)		
Gender	Male	54637 (67.07)	3278 (81.68)	120 (58.54)	58035 (67.74)	381.59	<0.001
	Female	26822 (32.93)	735 (18.32)	85 (41.46)	27642 (32.26)		
Household registration	The city	58205 (71.45)	2885 (71.89)	117 (57.07)	61207 (71.44)	21.15	<0.001
	Other cities	23254 (28.55)	1128 (28.11)	83 (42.93)	24470 (28.56)		
Educational level*	Primary school	26337 (32.33)	1225 (30.53)	34 (16.59)	27596 (32.21)	3448.98	<0.001
	Junior or high school	17825 (21.88)	2330 (58.06)	152 (74.15)	20307 (23.70)		
	College and above	124 (0.15)	6 (0.15)	2 (0.98)	132 (0.15)		
	Preschool/illiterate	37172 (45.64)	452 (11.26)	17 (8.29)	37641 (43.93)		
Occupation	Preschool children	37158 (45.62)	452 (11.26)	14 (6.83)	37624 (43.91)	3056.84	<0.001
	School students	40499 (49.72)	2873 (71.59)	126 (61.46)	43498 (50.77)		
	Staff or workers	2683 (3.29)	414 (10.32)	27 (13.17)	3124 (3.65)		
	Others	1119 (1.37)	274 (6.83)	38 (18.54)	1431 (1.67)		
Age group, years old	0~2	17788 (21.84)	164 (4.09)	5 (2.44)	17957 (20.96)	3754.25	<0.001
	3~5	18702 (22.96)	271 (6.75)	10 (4.88)	18983 (22.16)		
	6~11	24302 (29.83)	973 (24.25)	23 (11.22)	25298 (29.53)		
	12~14	8581 (10.53)	900 (22.43)	32 (15.61)	9513 (11.10)		
	15~17	12086 (14.84)	1705 (42.49)	135 (65.85)	13926 (16.25)		
Week of injuries occur	Weekend	24378 (29.93)	1169 (29.13)	67 (32.68)	25614 (29.90)	1.92	0.383
	Working day	57081 (70.07)	2844 (70.87)	138 (67.32)	60063 (70.10)		
Time that injuries occurred (o’clock)	0~3	2482 (3.05)	401 (9.99)	32 (15.61)	2915 (3.40)	718.60	<0.001
	4~7	2092 (2.57)	138 (3.44)	11 (5.37)	2241 (2.62)		
	8~11	15320 (18.81)	645 (16.07)	22 (10.73)	15987 (18.66)		
	12~15	15769 (19.36)	697 (17.37)	32 (15.61)	16498 (19.26)		
	16~19	27667 (33.96)	1319 (32.87)	37 (18.05)	29023 (33.87)		
	20~23	18129 (22.26)	813 (20.26)	71 (34.63)	19013 (22.19)		
Time of doctor visit for children with injuries (o’clock)	0~3	2991 (3.67)	457 (11.39)	39 (19.02)	3487 (4.07)	911.38	<0.001
	4~7	1366 (1.68)	158 (3.94)	13 (6.34)	1537 (1.79)		
	8~11	11254 (13.82)	358 (8.92)	19 (9.27)	11631 (13.58)		
	12~15	16028 (19.68)	696 (17.34)	32 (15.61)	16756 (19.56)		
	16~19	24184 (29.69)	1108 (27.61)	29 (14.15)	25321 (29.55)		
	20~23	25636 (31.47)	1236 (30.80)	73 (35.61)	26945 (31.45)		
Place where injury occurred	At home	34556 (42.42)	621 (15.47)	100 (48.78)	35277 (41.17)	2449.42	<0.001
	Public place	29840 (36.63)	2624 (65.39)	73 (35.61)	32537 (37.98)		
	On the road	13945 (17.12)	308 (7.68)	8 (3.90)	14261 (16.65)		
	Workplace	2706 (3.32)	342 (8.52)	14 (6.83)	3062 (3.57)		
	Others	412 (0.51)	118 (2.94)	10 (4.88)	540 (0.63)		
Nature of the injury^	Fracture	7180 (8.81)	91 (2.27)	18 (8.78)	7289 (8.51)	1183.51	<0.001
	Sprain/strain	3631 (4.46)	39 (0.97)	7 (3.41)	3677 (4.29)		
	Sharp wound/bites /open wound	26725 (32.81)	1362 (33.94)	89 (43.41)	28176 (32.89)		
	Bruise	34493 (42.34)	2266 (56.47)	32 (15.61)	36791 (42.94)		
	Burns	4521 (5.55)	16 (0.40)	2 (0.98)	4539 (5.30)		
	Concussion/brain contusion	2272 (2.79)	122 (3.04)	3 (1.46)	2397 (2.80)		
	Organ system injuries	973 (1.19)	61 (1.52)	34 (16.59)	1068 (1.25)		
	Others	1663 (2.04)	56 (1.40)	20 (9.76)	1739 (2.03)		
Activities at the time of the injuries	Working	6325 (7.76)	83 (2.07)	15 (7.32)	6423 (7.50)	1200.48	<0.001
	Leisure	61286 (75.24)	3106 (77.40)	117 (57.07)	64509 (75.29)		
	Housework/learning	1292 (1.59)	81 (2.02)	1 (0.49)	1374 (1.60)		
	Sports	2020 (2.48)	323 (8.05)	6 (2.93)	2349 (2.74)		
	Driving	5711 (7.01)	7 (0.17)	4 (1.95)	5722 (6.68)		
	Others	4825 (5.92)	413 (10.29)	62 (30.24)	5300 (6.19)		
Injured body part	Head	30288 (37.18)	1648 (41.07)	27 (13.17)	31963 (37.31)	2576.13	<0.001
	Upper limbs	24624 (30.23)	684 (17.04)	103 (50.24)	25411 (29.66)		
	Lower limbs	16465 (20.21)	321 (8.00)	11 (5.37)	16797 (19.61)		
	Trunk	4365 (5.36)	659 (16.42)	9 (4.39)	5033 (5.87)		
	Multiple parts/body wide	4629 (5.68)	621 (15.47)	12 (5.85)	5262 (6.14)		
	Others	1088 (1.34)	80 (1.99)	43 (20.98)	1211 (1.41)		
Severity	Mild	65059 (79.87)	3197 (79.67)	119 (58.05)	68375 (79.81)	86.92	<0.001
	Moderate	15975 (19.61)	777 (19.36)	80 (39.02)	16832 (19.65)		
	Severe	425 (0.52)	39 (0.97)	6 (2.93)	470 (0.55)		
Ending	Going home after treatment	69827 (85.72)	3370 (83.98)	133 (64.88)	73330 (85.59)	146.83	<0.001
	Observing/hospitalization/transfer to another hospital	10159 (12.47)	583 (14.53)	67 (32.68)	10809 (12.62)		
	Death	25 (0.03)	2 (0.05)	2 (0.98)	29 (0.03)		
	Others	1448 (1.78)	58 (1.45)	3 (1.46)	1509 (1.76)		
Mechanisms of injuries	Falls	41030 (50.37)	170 (4.24)	29 (14.15)	41229 (48.12)	14311.63	<0.001
	Animal bites	11,247 (13.81)	683 (17.02)	6 (2.93)	11936 (13.93)		
	Road traffic injuries	9790 (12.02)	13 (0.32)	9 (4.39)	9812 (11.45)		
	Blunt injuries	7214 (8.86)	2385 (59.43)	25 (12.20)	9624 (11.23)		
	Knife/sharp injuries	5292 (6.50)	468 (11.66)	76 (37.07)	5836 (6.81)		
	Burns	4551 (5.59)	15 (0.37)	2 (0.98)	4568 (5.33)		
	Poisoning	332 (0.41)	22 (0.55)	48 (23.41)	402 (0.47)		
	Firearm injuries	45 (0.06)	3 (0.07)	0	48 (0.06)		
	Suffocation	21 (0.03)	2 (0.05)	0	23 (0.03)		
	Drowning	13 (0.02)	2 (0.05)	1 (0.49)	16 (0.02)		
	Sexual invasion	0	9 (0.22)	0	9 (0.01)		
	Others	1924 (2.36)	241 (6.01)	9 (4.39)	2174 (2.54)		
Total		81459 (100)	4013 (100)	205 (100)	85677 (100)	/	/

Note: n = number of cases; % = composition ratio.

* ^1Unintentional injuries missing.

### Modelling to discriminate intentional from unintentional injuries based on Machine Learning

The contribution of predictors of mechanisms of injuries, educational level, occupation, age group, places where injury occurred, injured body part, activities at the time of injury, and the nature of the injury was 0.0955, 0.0401, 0.0377, 0.0357, 0.0271, 0.0202, 0.0165, and 0.0121, respectively. The other 8 predictive variables with a contribution index <0.007 were removed from the model to reduce redundant features during modelling and improve the program running efficiency (Table 2).

**Table 2:** The contribution of predictors.

Rank	Variables	Information entropy	Rank	Variables	Information entropy
1	Mechanisms of injuries	0.0955	9	Gender	0.0068
2	Educational level	0.0401	10	Time of doctor visit for children with injuries	0.0067
3	Occupation	0.0377	11	Time that injuries occurred	0.0063
4	Age group	0.0357	12	Region	0.0056
5	Place where injury occurred	0.0271	13	Ending	0.0016
6	Injured body part	0.0202	14	Severity	0.0012
7	Activities at the time of the injuries	0.0165	15	Week of injuries occur	0.0009
8	Nature of the injury	0.0121	16	Household registration	0.0003

The accurate probability of discrimination for true value = 1 (unintentional injuries) by the Adaboost and DNN models was 0.980 and 0.977, respectively; the NB model had the highest accuracy (0.502) for the true value of the intention of injury = 2 (self-harm/suicide) and the highest accuracy (0.658) with the true value = 3 (violent attacks), but the accuracy (0.925) with the true value = 1 (unintentional injuries) was lowest (Table 3).

**Table 3:** Confusion matrix of intention of injury discrimination.

		1	2	3
**NB**	1	0.925*	0.015*	0.061*
	2	0.312^&^	0.502^&^	0.185^&^
	3	0.306	0.035	0.658
**DT**	1	0.976	0.008	0.016
	2	0.473	0.463	0.063
	3	0.428	0.032	0.541
**RF**	1	0.976	0.005	0.018
	2	0.546	0.351	0.102
	3	0.406	0.023	0.570
**Adaboost**	1	0.980	0.006	0.014
	2	0.532	0.400	0.068
	3	0.477	0.022	0.501
**DNN**	1	0.977	0.007	0.017
	2	0.473	0.434	0.093
	3	0.414	0.033	0.553

NOTE: 1 = Unintentional injuries, 2 = Self-harm/suicide, 3 = Violent attacks.

**Note**:

*When the intention of injury true value = 1 (unintentional injuries), the NB model had a probability of 0.925 to judge correctly, had the probability of 0.015 to discriminate incorrectly as self-harm/suicide, and had the probability of 0.061 to discriminate incorrectly as violent attacks.

^&^When the intention of injury true value = 2 (self-harm/suicide), the NB model has a probability of 0.502 to judge correctly, the probability of 0.312 to discriminate incorrectly as unintentional injuries and the probability of 0.185 to discriminate incorrectly as violent attacks.

**Fig 1:**
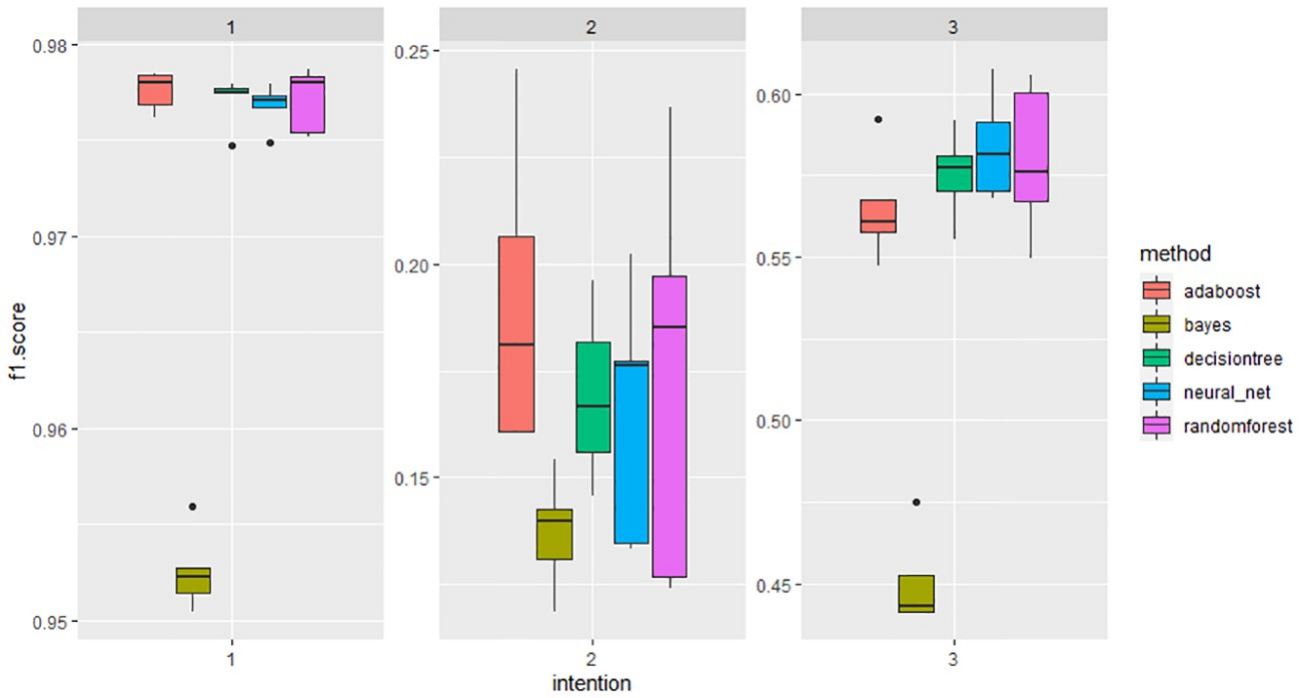
NB, DT, RF, Adaboost, DNN model F1- score for 5-fold to determine the intention of injury. NOTE: 1 = Unintentional injuries, 2 = Self-harm/suicide, 3 = Violent attacks.

The RF and Adaboost models had the highest accuracy (0.956), followed by the DNN and DT models (0.955), and the NB model (0.911). For the precision, in general, when the intention of injury = 1, the positive predictive value tended to be predicted correctly, and the intention of injury = 2/3 was opposite, which was related to the imbalance of classification of the intention of injury in the data. For the recall, the sensitivity of the DNN model was higher relatively. The F1-score of the five models was >0.950 when the intention of injury = 1, but they were lower when the intention of injury = 2/3 (Table 4 and Fig 1).

**Table 4:** Model evaluation indicators.

	Accuracy	Precision			Recall			F1- score		
		1	2	3	1	2	3	1	2	3
NB	0.911	0.983	0.072	0.346	0.925	0.502	0.658	0.953	0.126	0.453
DT	0.955	0.978	0.109	0.624	0.976	0.463	0.541	0.977	0.177	0.579
RF	0.956	0.979	0.120	0.603	0.976	0.351	0.570	0.978	0.179	0.586
Adaboost	0.956	0.975	0.127	0.632	0.980	0.400	0.501	0.978	0.193	0.559
DNN	0.955	0.978	0.116	0.616	0.977	0.434	0.553	0.977	0.183	0.583

NOTE: 1 = Unintentional injuries, 2 = Self-harm/suicide, 3 = Violent attacks.

**Fig 2:**
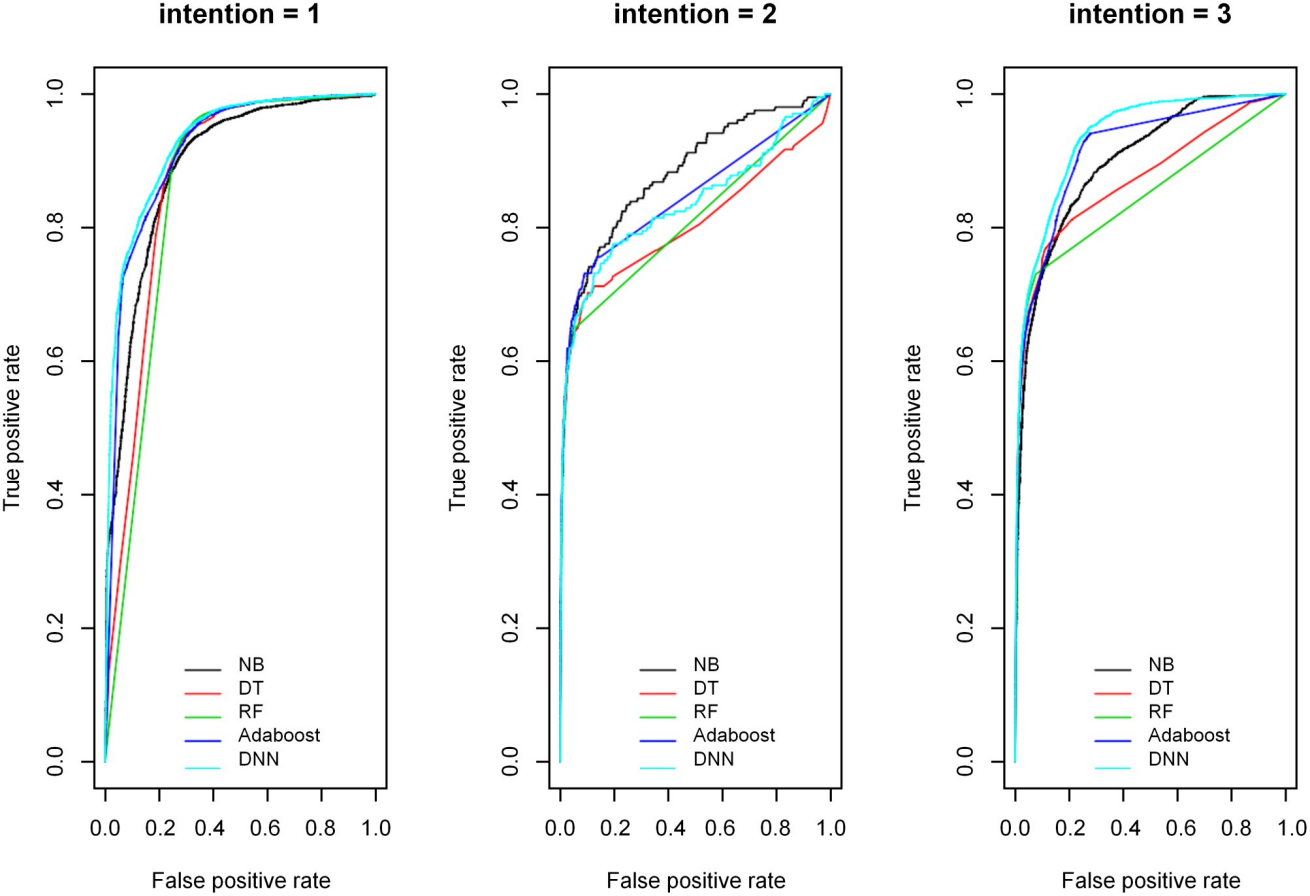
NB, DT, RF, Adaboost, DNN model ROC curve to determine the intention of injury. NOTE: 1 = Unintentional injuries, 2 = Self-harm/suicide, 3 = Violent attacks.

For the area under the ROC curve with intention of injury = 1/2/3, the NB model was 0.891, 0.880, and 0.897, respectively; the DT model was 0.870, 0.803, and 0.871, respectively; the RF model was 0.850, 0.809, and 0.845, respectively; the Adaboost model was 0.914, 0.846, and 0.914, respectively; the DNN model was 0.927, 0.835, and 0.934, respectively. Comparing the five models, the DNN and Adaboost models had higher predictive values for intention of injury determination (Fig 2).

### Model test

The cases with the intention of injury = 4/5 (unclear/others) were judged by the five models. The Consistency rate between the DT and Adaboost models was 96.83%, between DT and DNN models was 94.61%, and between Adaboost and DNN models was 94.41% (Table 5). The average percentage judged by the five models for the intention of injury = 1/2/3 was accounting for 86.57%, 6.81%, and 6.62%, respectively.

**Table 5:** The consistency rate (%) of comparison of NB, DT, RF, Adaboost, DNN models to determine the intention of injury.

	NB	DT	RF	Adaboost	DNN
NB	1	86.69	84.27	86.99	86.85
DT	86.69	1	94.33	96.83	94.61
RF	84.27	94.33	1	94.33	93.29
Adaboost	86.99	96.83	94.33	1	94.41
DNN	86.85	94.61	93.29	94.41	1

## Discussion

### The significance of the study on the intention of injury

Study has shown that unintentional injuries are the most common cause of injury-related deaths (57.00%) among children and adolescents, and 43.00% of injuries were intentional injury [12]. Studies in Pakistan have shown that intentional injuries account for 8.20% of the outpatient/emergency injuries of children ≤18 years old [13]. South Korea’s outpatient /emergency treatment injury study captured 10.50% of intentional injuries [8]. Gallaher JR et al. [14] analysed all injuries in a traumatic centre in Malawi <18 years old, showing that intentional injuries accounted for 8.10%. In this study, only 4.88% of injury cases among children and adolescents were intentional injuries as judged by doctors; another 0.83% of injury cases were others/unclear as for the intention of injury. Compared with other countries and studies, this study captured low levels of intentional injuries in the outpatient / emergency treatment population among children and adolescents. The possible reasons were that if the patient did not seek medical help after the injury, it was difficult to collect cases of injuries, especially self-harm/suicide; children and adolescents seeking medical help may intentionally conceal the true intention of their injury and may self-report the injury as an unintentional injury because of the stigma of the intentional injury, resulting in misclassification of injuries; some violent abuses, for sensitive reasons, may be reported by guardians as unintentional injuries to hide potential criminal behaviour, leading to misclassification bias.

Studies showed that children between the ages of 6 and 16 were often subjected to physical attacks [10], and teenagers may experience school-based violence or bullying [15]. In addition, in cases of child abuse, it was often claimed that the injury was caused by an accident [16]. Child sexual abuse in China has been a common and serious problem, with a combined incidence of childhood sexual abuse of 18.20% [17]. Although there are children suspected of having traumatic wounds that have been caused by violent attacks, research on the intent of these injuries is relatively lacking in the literature. In many countries, the true severity of intentional injury problems has been greatly underestimated. Data often come from passive reporting by health systems with the possibility of underreporting or misreporting, and there are other general misclassifications of the intention of injury.

The accurate discrimination of the intentions of injury intentions is of great significance. First, it helps to detect potential intentional injuries in children and adolescents in a timely manner, such as abuse, campus bullying, and self-harm/suicide. Second, it helps to provide timely implementation of case tracking, of finding illegal activities, of psychological counselling for children and adolescents, and of peer education. Third, it reduces the occurrence of intentional injury behaviours of children and adolescents, protects children’s physical and mental health, and achieves effective ways to protect children’s rights and sustainable development goals. Finally, it is of great significance for reducing the burden of diseases such as death and disability caused by intentional injury to children and adolescents, and even to promote physical and mental health after adulthood.

### The Machine Learning application of discriminants of the intention of injury

In terms of injury-related research, the ML model was developed to help identify injury cases in narrative texts and classify the mechanisms that cause injuries in a timelier manner [18]. In addition, studies have used ML algorithms to predict injury outcomes and burn mortality [19, 20]. In terms of the discrimination of the intent of injury, Paek SH et al. [21] reported a relatively low rate of suspected child abuse and developed a child abuse screening tool called “FIND”. The Influencing factors of “FIND” included physical examination, contradictory injury mechanisms, delayed visits, inappropriate guardianship, poor child hygiene, and long-term bone injuries in the head or other parts of the child. Kim PT et al. [22] developed a logistic model to assess potential predictors of increased risk of intentional injury. It was found that 1/4 of infants with head injuries in public places and no witnesses were identified as victims of intentional injury. This model was used to routinely screen high-risk populations to avoid missing intentional injuries. Bousema S et al. [23] screened suspicious child abuse in children with burns and confirmed that 9.00% were suspected of abusing or neglecting children. However, as of now, no relevant research reports using an ML algorithm to judge the intention of injury and mine potential intentional injuries had been found. This study hoped to establish a model through ML to discriminate the intention of injury, to reduce the false positive rate of intention of injury, and to effectively avoid the misclassification. Model discrimination can also exploit potential intentional injuries of children and adolescents.

### Injury intention discrimination modelling

For the mechanisms of injuries, study has shown that among adolescents, 61% of intentional firearm deaths resulted from homicide and 98% of intentional suffocation deaths resulted from suicide [12]. Studies of intentional injury in adolescents indicated that self-inflicted poisoning was common in adolescents and was a risk factor for suicide [24, 25]. In addition, the most common methods of self-harm/suicide were cuts and poisoning; blunt injuries mainly involved violent attacks. For the nature of injury, some studies found that soft tissue damage was most common in violent attacks, and cuts and fractures were more common in self-harm [16]. Gallaher JR et al. [14] showed that the average age of intentional injury patients was than that of patients with unintentional injuries, with intentional injuries reported more often in men than women; among intentional injuries, there were more injuries at night and more injuries with soft tissue damage, with head injury the most common cause of death. Studies had also shown that intentional injuries in rural students was higher than in urban students [7]. Therefore, based on the feature extraction of the influencing factors of intention of injury, this study found that there were differences in the intentions of injury of children and adolescents of different age groups, genders, regions, household registration, injury body sites, etc. The mechanisms of injury were different with the different intentions of injury. For example, children and adolescents who committed suicide often chose to cut the upper limbs with sharp objects, and blunt instruments were often used to hit the victim’s head when violent attacks happened. Therefore, the model included 16 variables in the very beginning. To reduce redundant features and to improve program operational efficiency, the model described the factors with the most influence on the intention of injury, and the 8 predictive variables with a lower contribution were removed from the model. The mechanisms of injury and the patient educational level, occupation, age group, and the place of injury were used as predictors.

The NB, DT, RF, Adaboost and DNN models in the ML algorithm were used to establish the model to discriminate the intentions of injury. The discrimination of the intentions of injury was a multi-classification that used various variables as the influencing factors to predict the three kinds of intentions of injury. The influencing factors were all categorical variables. In this study, the classical algorithms in ML were suitable for discriminative modelling. For example, NB was suitable for modelling categorical variables. DT constructed a sequence of trees, each of which learned to compensate for the errors left by the previous tree and got the classifier. RF and Adaboost were comprehensive lifting algorithms to integrate multiple weak classifiers. DNN combines low-level features to form a more abstract high-level to represent attribute categories or features. After comparing the model evaluation indicators, DNN and Adaboost models had a good discriminating ability for determining the intention of injury.

The intentions of injury in this study were unbalanced. If unbalanced data were used to model directly, then the discriminant results would easily be biased towards a larger number of categories. If the model cannot effectively identify the intentional damage, then the model is meaningless. The random sampling and processing were used for the unbalanced data, and thus the modelling effect tended to be good. The average proportion of unintentional injury, self-harm/suicide and violent attacks was 86.57%, 6.81%, and 6.62%, respectively using the model to determine the injury intentions of the cases for which intentions were unclear. The judged proportion of intentional injury in the study was similar to that of reported by Han H et al. [8], Amanullah S et al. [9], and Gallaher JR et al. [14] Whether the result of the determination of the intention of injury was in line with the actual situation needs to be further explored in future research.

### Limitations

There were many factors that affected the intentions of injuries, such as psychological and emotional factors, severe punishment of parents, low parental monitoring, parental migration patterns and parent-child attachment levels, and some factors that have not yet been recognized. This information was not included in the report, which theoretically reduced the performance of the model to discriminate the intentions of injury. Modeling with retrospective data mean that the test data was not verification, but the results of the percentage of intentional injuries derived from the test data were similar to other studies, and the verification model was our next research direction.

## Conclusion

This study used the ML algorithm to determine the intentions of injury in children and adolescents. It suggested that the DNN and Adaboost models had higher values for the discriminating the intentions of injury. It was expected to transform the model into a tool for rapid diagnosis and to further discriminate the intention of injury through the model and to explore the potential intentional injury of children and adolescents.

## Supporting information

S1 Dataset (XLSX)

## Abbreviations

ACEs: Adverse childhood experiences
DNN: Deep Neural Networks
DT: Decision Tree
ML: Machine Learning
NB: Naive Bayes
NISS: Chinese National Injury Surveillance System
RF: Random Forest
ROC: Receiver Operating Characteristic

## References

[1] Somer O, Bildik T, Kabukçu-Başay B, Güngör D, Başay Ö, Farmer RF. Prevalence of non-suicidal self-injury and distinct groups of self-injurers in a community sample of adolescents. Soc Psychiatry Psychiatr Epidemiol. 2015;50(7):1163–1171. 10.1007/s00127-015-1060-z . 25952581

[2] Hughes K, Bellis MA, Hardcastle KA, Sethi D, Butchart A, Mikton C, et al The effect of multiple adverse childhood experiences on health: a systematic review and meta-analysis. Lancet Public Health. 2017;2(8):e356–e366. 10.1016/S2468-2667(17)30118-4 . 29253477

[3] Global Burden of Disease Pediatrics Collaboration, Kyu HH, Pinho C, Wagner JA, Brown JC, Bertozzi-Villa A, et al Global and National Burden of Diseases and Injuries Among Children and Adolescents Between 1990 and 2013: Findings From the Global Burden of Disease 2013 Study. JAMA Pediatr. 2016;170(3):267–287. 10.1001/jamapediatrics.2015.4276 . 26810619 PMC5076765

[4] Hillis S, Mercy J, Amobi A, Kress H. Global Prevalence of Past-year Violence Against Children: A Systematic Review and Minimum Estimates. Pediatrics. 2016;137(3):e20154079 10.1542/peds.2015-4079 26810785 PMC6496958

[5] Stoltenborgh M, van Ijzendoorn MH, Euser EM, Bakermans-Kranenburg MJ. A global perspective on child sexual abuse: meta-analysis of prevalence around the world. Child Maltreat. 2011;16(2):79–101. 10.1177/1077559511403920 21511741

[6] Stoltenborgh M, Bakermans-Kranenburg MJ, van Ijzendoorn MH, Alink LR. Cultural-geographical differences in the occurrence of child physical abuse? A meta-analysis of global prevalence. Int J Psychol. 2013;48(2):81–94. 10.1080/00207594.2012.697165 23597008

[7] Wang Y, Zhang M, Chen H. Self-Injury Among Left-Behind Adolescents in Rural China: The Role of Parental Migration and Parent-Child Attachment. Front Psychol. 2019;9:2672 Published 2019 Jan 7. 10.3389/fpsyg.2018.02672 30666226 PMC6330276

[8] Han H, Park B, Park Boh, Park N, Park JO, Ahn KO, et al The Pyramid of Injury: Estimation of the Scale of Adolescent Injuries According to Severity. J Prev Med Public Health. 2018;51(3):163–168. 10.3961/jpmph.18.027 29886712 PMC5996192

[9] Amanullah S, Heneghan JA, Steele DW, Mello MJ, Linakis JG. Emergency department visits resulting from intentional injury in and out of school. Pediatrics. 2014;133(2):254–261. 10.1542/peds.2013-2155 24420809

[10] Yuan W, Pengpeng Y, Leilei D. Characteristics of unintentional and intentional injuries among children from emergency and outpatient rooms in China, 2006–2014. Chin J Dis Control Prev. 2016;20, 670–674,743 10.16462/j.cnki.zhjbkz.2016.07.007.

[11] Klinke M, Schmidt CM, Tegtmeyer L, Reinshagen K, Boettcher M, Koenigs I. Undetected Cases of Non-Accidental Burns in Children—Preventive Strategies. Klin Padiatr. 2018;230(2):61–67. 29258160 10.1055/s-0043-119416

[12] Cunningham RM, Walton MA, Carter PM. The Major Causes of Death in Children and Adolescents in the United States. N Engl J Med. 2018;379(25):2468–2475. 10.1056/NEJMsr1804754 30575483 PMC6637963

[13] Khan U, Hisam B, Zia N, Mir M, Alonge O, Jamali S, et al Uncovering the burden of intentional injuries among children and adolescents in the emergency department. BMC Emerg Med. 2015;15 Suppl 2(Suppl 2):S6 10.1186/1471-227X-15-S2-S6 26692292 PMC4682402

[14] Gallaher JR, Wildfire B, Mabedi C, Cairns BA, Charles AG. Intentional injury against children in Sub-Saharan Africa: A tertiary trauma centre experience. Injury. 2016;47(4):837–841. 10.1016/j.injury.2015.10.072 26584730

[15] Gordon AR, Conron KJ, Calzo JP, White MT, Reisner SL, Austin SB. Gender Expression, Violence, and Bullying Victimization: Findings from Probability Samples of High School Students in 4 US School Districts. J Sch Health. 2018;88(4):306–314. 10.1111/josh.12606 29498058 PMC5836796

[16] Madea B, Banaschak S. Fatal child abuse, bodily injury followed by death or accidental fall? Arch Kriminol. 2015;236(1–2):11–30. 26399119

[17] Peng L, Zhang SH, Yang J, Li Y, Ye YF, Dong XM, et al Meta analysis on the incidence rates of child sexual abuse in China. Chin J Epidemiol. 2013;34(12):1245–1249 24518030

[18] Vallmuur K, Marucci-Wellman HR, Taylor JA, Lehto M, Corns HL, Smith GS. Harnessing information from injury narratives in the ’big data’ era: understanding and applying machine learning for injury surveillance. Inj Prev. 2016;22 Suppl 1(Suppl 1):i34–i42. 10.1136/injuryprev-2015-041813 26728004 PMC4852152

[19] Liu NT, Salinas J. Machine Learning for Predicting Outcomes in Trauma. Shock. 2017;48(5):504–510. 10.1097/SHK.0000000000000898 28498299

[20] Stylianou N, Akbarov A, Kontopantelis E, Buchan I, Dunn KW. Mortality risk prediction in burn injury: Comparison of logistic regression with machine learning approaches. Burns. 2015;41(5):925–934. 10.1016/j.burns.2015.03.016 25931158

[21] Paek SH, Jung JH, Kwak YH, Kim DK, Ryu JM, Noh H, et al Development of screening tool for child abuse in the korean emergency department: Using modified Delphi study. Medicine (Baltimore). 2018;97(51):e13724 10.1097/MD.0000000000013724 30572510 PMC6319994

[22] Kim PT, McCagg J, Dundon A, Ziesler Z, Moody S, Falcone RA Jr. Consistent screening of admitted infants with head injuries reveals high rate of nonaccidental trauma. J Pediatr Surg. 2017;52(11):1827–1830. 10.1016/j.jpedsurg.2017.02.014 28302360

[23] Bousema S, Stas HG, van de Merwe MH, Oen IM, Baartmans MG, van Baar ME, et al Epidemiology and screening of intentional burns in children in a Dutch burn centre. Burns. 2016;42(6):1287–1294. 10.1016/j.burns.2016.01.009 27211360

[24] Ulseth ET, Freuchen A, Köpp UMS. Acute poisoning among children and adolescents in southern Norway. Tidsskr Nor Laegeforen. 2019;139(13):10.4045/tidsskr.17.1116. 10.4045/tidsskr.17.1116 31556525

[25] Zainum K, Cohen MC. Suicide patterns in children and adolescents: a review from a pediatric institution in England. Forensic Sci Med Pathol. 2017;13(2):115–122. 10.1007/s12024-017-9860-y 28349246

